# Lower body mass index potentiates the association between skipping breakfast and prevalence of proteinuria

**DOI:** 10.3389/fendo.2022.916374

**Published:** 2022-08-19

**Authors:** Jun Muratsu, Kei Kamide, Takashi Fujimoto, Yasushi Takeya, Ken Sugimoto, Yoshiaki Taniyama, Atsuyuki Morishima, Katsuhiko Sakaguchi, Hiromi Rakugi

**Affiliations:** ^1^ Department of Nephrology and Hypertension, Sumitomo Hospital, Osaka, Japan; ^2^ Department of Nephrology, Rinku General Medical Center, Izumisano City, Japan; ^3^ Division of Health Sciences, Osaka University Graduate School of Medicine, Osaka, Japan; ^4^ Department of Geriatric and General Medicine, Osaka University Graduate School of Medicine, Osaka, Japan; ^5^ Department of General and Geriatric Medicine, Kawasaki Medical School General Medical Center, Okayama, Japan

**Keywords:** skipping breakfast, proteinuria, body mass index, health checkup, waist circumference

## Abstract

**Background:**

Proteinuria is an important predictor of cardiovascular disease and mortality. Several studies reported the association between skipping breakfast and the prevalence of proteinuria. Furthermore, skipping breakfast was associated with an increased risk of obesity. Although proteinuria is highly prevalent in obese individuals, the association between the prevalence of proteinuria and low body mass index (BMI) was reported in a previous cross-sectional study in asymptomatic individuals without known kidney diseases. The aim of this cross-sectional study was to assess the clinical impact of BMI on the association between skipping breakfast and the prevalence of proteinuria in normal renal function subjects.

**Methods:**

The present study included 26,888 subjects (15,875 males and 11,013 females) with an estimated glomerular filtration rate ≥60 ml/min/1.73 m^2^ and no history of kidney disease who underwent a health checkup in Sumitomo Hospital. The association between skipping breakfast and the prevalence of proteinuria (defined as dipstick proteinuria of ≥1+) was assessed using logistic regression models adjusted for clinically relevant factors.

**Results:**

Skipping breakfast was reported in 3,306 males (20.8%) and 1,514 females (13.8%). Multivariable adjusted logistic regression models showed that skipping breakfast was significantly associated with the prevalence of proteinuria above 1+. This association was evident in lower BMI subjects, even after adjusting for clinically relevant factors (adjusted odds ratios of males and females were 1.67 [1.17–2.38] and 1.92 [1.31–2.82], respectively), whereas this association was not evident in higher BMI subjects.

**Conclusion:**

Lower BMI subjects with proteinuria might need to be careful about skipping breakfast.

## Introduction

Patients with chronic kidney disease (CKD) have a high risk of end-stage kidney disease (ESKD) (1), cardiovascular disease (CVD) (2), and mortality (3). CKD is characterized by proteinuria and/or low glomerular filtration rate (GFR) (4). Independent of estimated GFR (eGFR), proteinuria is an important predictor of ESKD (5), CVD, and mortality (6).

In previous reports, proteinuria was associated with various unhealthy life behaviors (7–11). Among the unhealthy life behaviors, the association between skipping breakfast and proteinuria was reported (12). A retrospective cohort study of 10,133 university workers reported that skipping breakfast was a risk factor for the incidence of proteinuria in females, but not in males (13). Previously, a systematic review including 36 cross-sectional and 9 cohort studies indicated that skipping breakfast increased the risk of obesity (14). In addition, a systematic review and meta-analysis of a prospective cohort study indicated that skipping breakfast was associated with an increased risk of type 2 diabetes, and the association was mediated by body mass index (BMI) (15). Although proteinuria is highly prevalent in obese individuals, the association between the prevalence of proteinuria and low BMI was reported in a previous cross-sectional study (16).

The aim of this cross-sectional study was to assess the clinical impact of BMI on the association between skipping breakfast and the prevalence of proteinuria in 15,875 males and 11,013 females with normal renal function.

## Methods

### Study Population

**Figure 1:**
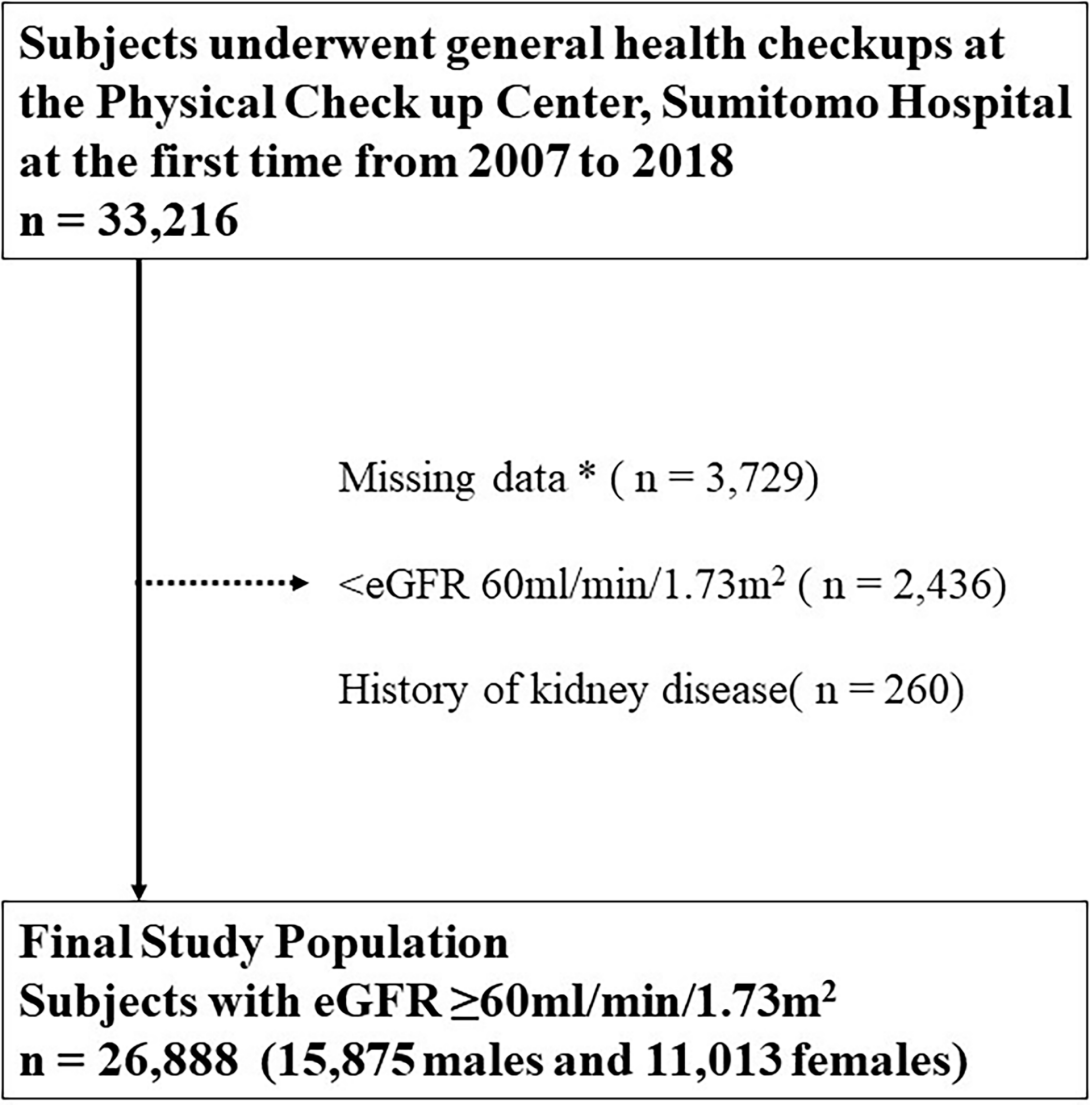
Inclusion and exclusion processes in the present study. * Including age, sex, body mass index, blood pressure, hemoglobin A1c, cholesterol, hemoglobin, aspartate transaminase, alanine aminotransferase, albumin, total cholesterol, triglyceride, high-density lipoprotein cholesterol, low-density lipoprotein cholesterol, fasting blood sugar level, insulin, uric acid, serum creatinine, eGFR, smoking status, drinking frequency, sleeping duration, exercise frequency, presence or absence of snacking and late-night dinner, and current treatments for diabetes, dyslipidemia, hypertension, hyperuricemia, stroke, and coronary disease. eGFR, estimated glomerular filtration rate.

Eligible subjects were 33,216 subjects from the general population who underwent a health checkup at the Physical Checkup Center of Sumitomo Hospital between April 2008 and December 2018. The health checkup program is designed to detect diseases at their early stages. Exclusion criteria included the following: not completing the questionnaire and missing data (n = 3,729, 11.2%), eGFR < 60 ml/min/1.73 m^2^ (n = 2,436, 7.3%), and a history of kidney disease (n = 260, 0.8%). This study included 26,888 (80.9%) subjects with normal renal function (eGFR ≥ 60 ml/min/1.73 m^2^), no history of kidney disease, and no missing data (**Figure 1**).

This study was approved by the human ethics committees of Sumitomo Hospital and was conducted according to the principles of the Declaration of Helsinki (approval No. 2020-14). Written informed consent to provide medical information and blood samples was obtained before the checkup examinations from all participants, and each subject had the right to refuse the use of their results.

### Measurements

The physical and laboratory data of 26,888 subjects at their first visit during the study period included age, sex, body mass index (BMI = weight [kg]/height^2^ [m^2^]), blood pressure, hemoglobin A1c (HbA1c), cholesterol, hemoglobin, aspartate transaminase, alanine aminotransferase, albumin, total cholesterol, triglyceride, high-density lipoprotein cholesterol, low-density lipoprotein cholesterol, fasting blood sugar (FBS) level, insulin, uric acid, serum creatinine, and eGFR.

The life behaviors for skipping breakfast, smoking, heavy alcohol intake, lack of exercise habits, short sleep duration, snacking, and late-night dinner as well as medical histories for hypertension, diabetes mellitus, dyslipidemia, stroke, hyperuricemia, and coronary artery disease were evaluated using standardized self-administered questionnaires and interviews by doctors at the participants’ first visit during the study period. The subjects who skipped breakfast over 3 days/week were defined as the skipping breakfast group. Sleep duration was categorized into <6, 6–8, and >8 h. Exercise habits were categorized into over 3 days/weeks, 1–2 days/weeks, and none. Alcohol amount per day was categorized into over 60, 40–60, 20–40, and 0–20 g of ethanol. One standard drink was defined as 500 ml of beer, 180 ml of Japanese sake (a traditional Japanese alcoholic beverage), 80 ml of shochu (a Japanese liquor), 60 ml of whiskey, or 240 ml of wine. The ethanol content per one standard drink was calculated to be equivalent to 20 g (17). Smoking status was classified as current smoking, past smoking, and never.

In Japan, the measurement method for HbA1c has changed from the Japan Diabetes Society (JDS) to National Glycohemoglobin Standardization Program (NGSP); hence, all HbA1c (JDS) data were converted from JDS to NGSP according to the guidelines of the JDS, as follows: HbA1c (NGSP) (%) = 1.02 × HbA1c (JDS) (%) + 0.25% (18). To calculate eGFR, the Japanese formula was used (eGFR [ml/min/1.73 m^2^] = 194 × serum creatinine [mg/dl] − 1.094 × Age [years] − 0.287 × 0.739 [if female]) (19). The homeostasis model assessment for insulin resistance (HOMA-IR) and beta-cell function (HOMA-β) are often used in clinical practice as an index of insulin resistance and are calculated as follows: HOMA-IR = fasting blood sugar (mg/dl) × IRI/405, and HOMA-β (%) = [360 × fasting insulin (mIU/L)]/[fasting blood sugar (mg/dl) – 63] (20).

The outcome measure of interest was proteinuria (defined as dipstick urinary protein ≥ 1+) using random spot urine samples. The results of the urine dipstick tests of proteinuria using MEDITAPE II 9U (Sysmex, Kobe, Japan) were measured using UX-2000 (ARKRAY Factory, Shiga, Japan) and recorded as negative, ±, 1+, 2+, or 3+. In UX-2000, urine qualitative analysis was performed by the color reaction of test paper from reflection photometry. The tests were conducted under the following conditions: the last food was consumed 14 h or more and fluids 3 h or more before the health checkup.

### Statistical Analysis

Baseline characteristics between skipping breakfast and taking breakfast were compared using ANOVA, χ^2^ test, *t*-test, or the Kruskal–Wallis test or the Wilcoxon rank-sum test, as appropriate.

The association between skipping breakfast and prevalence of proteinuria (dipstick proteinuria of ≥1+) was assessed using univariable and multivariable logistic regression models adjusting for the variables, including age, sex, BMI (kg/m^2^), FBS level, smoking status (current smoking, past smoking, or never), presence or absence of snacking and late-night dinner, and current treatments for diabetes, dyslipidemia, hypertension, hyperuricemia, stroke, or coronary disease. In previous reports, skipping breakfast is a marker of various unhealthy lifestyles (21). Therefore, we analyzed in multivariable logistic regression models adjusting for additional variables, including drinking frequency (ethanol amount: over 60, 40–60, 20–40, or 0–20 g), sleeping duration (<6, 6–8, or >8 h), and exercise frequency (over 3 days/weeks, 1–2 days/weeks, or none).

To clarify the effect of BMI and waist circumference on the relationship between skipping breakfast and the prevalence of proteinuria, BMI and waist circumference were studied in three groups with each 33rd percentile and 66th percentile by gender. The association of skipping breakfast with the prevalence of proteinuria was assessed in three subgroups with BMI (males <22.2, 22.2–24.5, and ≥24.5; females <19.3, 19.3–21.6, and ≥21.6 kg/m^2^) and waist circumference (males <81.0, 81.0–88.0, and ≥88.0; females <73.0, 73.0–80.0, and 80.0 cm ).

Categorical variables were expressed as numbers (percentages), and continuous variables were shown as mean ± standard deviation or median (interquartile range), as appropriate. Statistical significance was set at *p* < 0.05, if not specified. All statistical analyses were performed using Stata, version 14.2 (Stata Corp., http://www.stata.com).

## Results

The study population was 15,875 males (mean age 49 ± 11 years) and 11,013 females (mean age 48 ± 12 years) stratified on the BMI levels in **Tables 1A**, **B**. In males, 1,089 (20.9%), 970 (18.8%), and 1,247 (22.6%) skipped breakfast with BMI < 22.2, 22.2 ≤ BMI < 24.5, and 24.5 ≤ BMI, respectively. In females, 559 (15.2%), 431 (12.4%), and 524 (13.6%) skipped breakfast with BMI < 19.3, 19.3 ≤ BMI < 21.6, and 21.6 ≤ BMI, respectively.

**Table 1A:** Clinical characteristics of 15,875 males stratified by body mass index (BMI) levels.

Parameters	BMI<22.2 n= 5,201(32.8%)	22.2 ≤BMI <24.5 n= 5,159(32.5%)	24.5 ≤BMI n= 5,515(34.7%)
Age (years)	49±11	50±11	49±10
Height (cm)	171.2±6.1	171.0±5.9	171.1±6.1
Weight (kg)	60.2±5.7	68.1±5.1	79.0±9.5
BMI (kg/m^2^)	20.5±1.3	23.3±0.7	27.0±2.5
Waist circumference (cm)	77.4±5.2	84.4±4.3	93.1±7.0
**Medical History, n (%)**	Hypertension	540 (10.4)	931 (18.1)	1,512 (27.4)
Diabetes mellitus	279 (5.4)	366 (7.1)	566 (10.3)
Dyslipidemia	614 (11.8)	950 (18.4)	1,345 (24.4)
Stroke	64 (1.2)	62 (1.2)	85 (1.5)
Hyperuricemia	295 (5.7)	447 (8.7)	727 (13.2)
Coronary artery disease	59 (1.1)	85 (1.7)	129 (2.3)
**Life-behavior, n (%)**	Skipping breakfast	1,089 (20.9)	970 (18.8)	1,247 (22.6)
Smoking habits	Current smoking	1,573 (30.2)	1,477 (28.6)	1,727 (31.3)
Past smoking	1,870 (36.0)	2,152 (41.7)	2,214 (40.2)
Never	1,758 (33.8)	1,530 (29.7)	1,574 (28.5)
Alcohol amount per day	Over 60 g	352 (6.8)	436 (8.5)	671 (12.2)
40-60g	996 (19.2)	1,136 (22.0)	1,308 (23.7)
20-40g	1,478 (28.4)	1,538 (29.8)	1,467 (26.6)
0-20g	2,375 (45.7)	2,049 (39.7)	2,069 (37.5)
Exercise habits	Over 3 days/weeks	948 (18.2)	994 (19.3)	907 (16.5)
1-2 days/weeks	1,738 (33.4)	1,899 (36.8)	1,975 (35.8)
None	2,515 (48.4)	2,266 (43.9)	2,633 (47.7)
Snacking	293 (5.6)	337 (6.5)	492 (8.9)
Late-night dinners	2,434 (46.8)	2,453 (47.6)	2,862 (51.9)
Sleeping duration (hour)
<6 hours	2,024 (38.9)	2,067 (40.1)	2,370 (43.0)
6-8 hours	3,076 (59.1)	3,015 (58.4)	3,068 (55.6)
>8 hours	101 (1.9)	77 (1.5)	77 (1.4)
**Physical findings on admission**			
Systolic blood pressure, mmHg	118±14	123±14	128±15
Diastolic blood pressure, mmHg	74±10	77±10	81±10
**Laboratory Data on admission**			
Hemoglobin, mg/dL	14.8±1.0	15.0±0.9	15.3±1.0
AST, unit/L	20 (17, 24)	21 (18, 26)	24 (19, 30)
ALT, unit/L	18 (14, 24)	21 (16, 29)	28 (20, 42)
Albumin, mg/dL	4.5±0.3	4.5±0.3	4.6±0.3
Total cholesterol, mg/dL	204±32	210±33	213±34
Triglyceride, mg/dL	86 (64, 121)	109 (80, 158)	136 (98, 196)
HDL-C, mg/dL	64 (55, 75)	58 (50, 67)	53 (46, 61)
LDL-C, mg/dL	117 (99, 138)	127 (108, 147)	132 (111, 152)
FBS, mg/dL	96±16	100±19	105±23
Creatinine, mg/dL	0.83±0.10	0.84±0.10	0.84±0.10
Uric acid, mg/dL	5.9±1.1	6.2±1.2	6.5±1.2
eGFR, mL/min/1.73m^2^	79.1 (71.7, 80.1)	76.8 (69.9, 85.2)	76.7 (69.4, 85.4)
Hemoglobin A1c (NGSP), %	5.5 (5.2, 5.7)	5.5 (5.3, 5.8)	5.6 (5.4, 6.0)
HOMA-beta	47.6 (34.7, 65.5)	58.3 (41.9, 79.6)	74.6 (52.5, 105.8)
HOMA-IR	0.93 (0.66, 1.28)	1.29 (0.92, 1.77)	1.93 (1.34, 2.81)
Proteinuria above (1+)	167 (3.2)	143 (2.8)	333 (6.0)

Categorical variables are expressed as numbers (percentages) and continuous variables are shown as mean ± standard deviation or median (interquartile range), as appropriate. BMI, body mass index; ALT, alanine aminotransferase; AST, aspartate transaminase; HDL, high-density lipoprotein; LDL, low-density lipoprotein; FBS, fasting blood sugar level; eGFR, estimated glomerular filtration rate.

**Table 1B:** Clinical characteristics of 11,013 females stratified by BMI levels.

Parameters		BMI < 19.3n = 3,685(33.5%)		19.3 ≤ BMI < 21.6n = 3,465(31.5%)	21.6 ≤ BMIn = 3,863(35.0%)
Age (years)		45 ± 11		48 ± 11	51 ± 12
Height (cm)		159.2 ± 5.4		158.5 ± 5.4	157.4 ± 5.8
Weight (kg)		45.8 ± 4.0		51.3 ± 3.8	60.5 ± 8.1
BMI (kg/m^2^)		18.0 ± 1.0		20.4 ± 0.6	24.4 ± 2.8
Waist circumference (cm)		69.8 ± 4.9		75.9 ± 5.0	85.6 ± 7.8
**Medical history, n (%)**					
Hypertension		140 (3.8)		224 (6.5)	604 (15.6)
Diabetes mellitus		45 (1.2)		49 (1.4)	154 (4.0)
Dyslipidemia		281 (7.6)		362 (10.5)	688 (17.8)
Stroke		13 (0.4)		21 (0.6)	44 (1.1)
Hyperuricemia		2 (0.1)		6 (0.2)	10 (0.3)
Coronary artery disease		14 (0.4)		20 (0.6)	45 (1.2)
**Life behavior, n (%)**					
Skipping breakfast		559 (15.2)		431 (12.4)	524 (13.6)
Smoking habits					
Current smoking		303 (8.2)		299 (8.6)	278 (7.2)
Past smoking		429 (11.6)		379 (10.9)	460 (11.9)
Never		2,953 (80.1)		2,787 (80.4)	3,125 (80.9)
Alcohol amount per day					
Over 60 g		67 (1.8)		60 (1.7)	73 (1.9)
40–60 g		199 (5.4)		190 (5.5)	207 (5.4)
20–40 g		475 (12.9)		498 (14.4)	543 (14.1)
0–20 g		2,944 (79.9)		2,717 (78.4)	3,040 (78.7)
Exercise habits					
Over 3 days/weeks		566 (15.4)		583 (16.8)	663 (17.2)
1–2 days/weeks		908 (24.6)		971 (28.0)	1,008 (26.1)
None		2,211 (60.0)		1,911 (55.2)	2,192 (56.7)
Snacking		694 (18.8)		740 (21.4)	1,083 (28.0)
Late-night dinners		763 (20.7)		722 (20.8)	873 (22.6)
Sleeping duration (hour)
<6 h		1,365 (37.0)		1,299 (37.5)	1,703 (44.1)
6–8 h		2,246 (61.0)		2,090 (60.3)	2,084 (54.0)
>8 h		74 (2.0)		76 (2.2)	76 (2.0)
**Physical findings on admission**						
Systolic blood pressure, mmHg		110 ± 14		114 ± 15	123 ± 17
Diastolic blood pressure, mmHg		68 ± 10		70 ± 10	76 ± 11
**Laboratory Data on admission**						
Hemoglobin, mg/dl		12.9 ± 1.2		12.9 ± 1.1	13.2 ± 1.2
AST, unit/L		19 (16, 22)		18 (16, 22)	19 (16, 23)
ALT, unit/L		13 (10, 17)		13 (10, 17)	16 (12, 22)
Albumin, mg/dl		4.5 ± 0.3		4.4 ± 0.3	4.4 ± 0.3
Total cholesterol, mg/dl		206 ± 37		210 ± 36	218 ± 38
Triglyceride, mg/dl		61 (48, 81)		69 (52, 93)	89 (64, 125)
HDL-C, mg/dl		78 (68, 89)		74 (64, 85)	67 (57, 77)
LDL-C, mg/dl		108 (90, 129)		115 (96, 137)	128 (107, 150)
FBS, mg/dl		88 ± 9		90 ± 10	96 ± 15
Creatinine, mg/dl		0.62 ± 0.08		0.62 ± 0.08	0.62 ± 0.08
Uric acid, mg/dl		4.2 ± 0.8		4.4 ± 0.9	4.8 ± 1.0
eGFR, ml/min/1.73 m^2^		82.1 (73.5, 92.7)		80.5 (72.3, 90.3)	78.7 (70.8, 88.6)
Hemoglobin A1c (NGSP), %		5.4 (5.2, 5.6)		5.4 (5.2, 5.7)	5.6 (5.3, 5.8)
HOMA-beta		56.6 (41.8, 75.3)		62.7 (46.3, 84.0)	73.1 (54.0, 99.9)
HOMA-IR		0.81 (0.60, 1.12)		0.98 (0.72, 1.33)	1.42 (0.99, 2.05)
Proteinuria above (1+)		161 (4.4)		103 (3.0)	123 (3.2)

Categorical variables are expressed as numbers (percentages), and continuous variables are shown as mean ± standard deviation or median (interquartile range), as appropriate.

BMI, body mass index; ALT, alanine aminotransferase; AST, aspartate transaminase; HDL, high-density lipoprotein; LDL, low-density lipoprotein; FBS, fasting blood sugar level; eGFR, estimated glomerular filtration rate.

In males, 167 (3.2%), 143 (2.8%), and 333 (6.0%) showed proteinuria above 1+ in BMI < 22.2, 22.2 ≤ BMI < 24.5, and 24.5 ≤ BMI, respectively. In females, 161 (4.4%), 103 (3.0%), and 123 (3.2%) showed proteinuria above 1+ in BMI < 19.3, 19.3 ≤ BMI < 21.6, and 21.6 ≤ BMI, respectively.

Of these, 3,306 males (20.8%) and 1,514 females (13.8%) skipped breakfast. In both males and females, subjects who skipped breakfast were of younger age and had a low prevalence of medical history. Compared with those who ate breakfast, those who skipped breakfast were likely to have a higher prevalence of being current smokers, drinking over 60 g of ethanol, snacking, and having late-night dinner and a low prevalence of exercise habits (**Supplementary Tables A**, **B**).

To assess the association between skipping breakfast and the prevalence of proteinuria, odds ratios were calculated using adjusted logistic regression models (**Tables 2A**, **B**). Unadjusted logistic regression models with proteinuria above 1+ showed significant associations in both males and females (*p* < 0.001). Even after clinically relevant factors (models 1 and 2) and additional unhealthy behavior variables (model 3) were adjusted, skipping breakfast had a significantly higher risk of proteinuria above 1+ in both male and female groups (adjusted odds ratios of males and females were as follows: model 1, 1.49 [1.24–1.78] and 1.83 [1.44–2.33]; model 2, 1.40 [1.16–1.68] and 1.75 [1.36–2.26]; and model 3, 1.36 [1.13–1.64] and 1.79 [1.38–2.31], respectively).

**Table 2A:** Logistic regression analysis for the prevalence of proteinuria above 1+ in males.

Males	Univariable Model		*Multivariable Model 1		**Multivariable Model 2		***Multivariable Model 3	
	Odds ratio(95% CI)	*p*-value	Odds ratio(95% CI)	*p*-value	Odds ratio(95% CI)	*p*-value	Odds ratio(95% CI)	*p*-value
Skipping breakfast	1.72	<0.001	1.49	<0.001	1.40	<0.001	1.36	0.001
	(1.45-2.05)		(1.24-1.78)		(1.16-1.68)		(1.13-1.64)	
BMI	1.11	<0.001	1.08	<0.001	1.06	<0.001	1.06	<0.001
	(1.09-1.13)		(1.06-1.10)		(1.03-1.08)		(1.03-1.08)	
Age	0.98	<0.001	0.97	<0.001	0.96	<0.001	0.96	<0.001
	(0.97-0.99)		(0.96-0.98)		(0.95-0.97)		(0.95-0.97)	
FBS	1.02	<0.001	1.02	<0.001	1.02	<0.001	1.02	<0.001
	(1.01-1.02)		(1.01-1.02)		(1.01-1.02)		(1.01-1.02)	

CI, confidence interval; BMI, body mass index; FBS, fasting blood sugar level.

*Adjusted for skipping breakfast, BMI (kg/m^2^), FBS (mg/dL) and age (y).

**Adjusted for model 1 + smoking status (none, past, vs. current), drinking ethanol amount(0-20 g, 20-40 g, 40-60 g, vs. over 60 g), and current treatment for hypertension, dyslipidemia, hyperuricemia, stroke, or coronary disease at their first visit during the study period.

***Adjusted for model 2 + sleep duration (<6 hours, 6-8 hours, vs. >8 hours), exercise habit weekly (over 3 days/weeks, 1-2 days/weeks, vs. none), snacking and late night dinner at their first visit during the study period.

**Table 2B:** Logistic regression analysis for the prevalence of proteinuria above 1+ in females.

Females	Univariable Model		*Multivariable Model 1		**Multivariable Model 2		***Multivariable Model 3	
	Odds ratio(95% CI)	*p*-value	Odds ratio(95% CI)	*p*-value	Odds ratio(95% CI)	*p*-value	Odds ratio(95% CI)	*p*-value
Skipping breakfast	2.17	<0.001	1.83	<0.001	1.75	<0.001	1.79	<0.001
	(1.72–2.75)		(1.44–2.33)		(1.36–2.26)		(1.38–2.31)	
BMI	0.98	0.291	0.98	0.212	0.97	0.129	0.97	0.080
	(0.95–1.02)		(0.95–1.01)		(0.94–1.01)		(0.94–1.00)	
Age	0.96	<0.001	0.96	<0.001	0.96	<0.001	0.96	<0.001
	(0.96–0.97)		(0.95–0.97)		(0.95–0.97)		(0.95–0.97)	
FBS	1.01	<0.001	1.02	<0.001	1.02	<0.001	1.02	<0.001
	(1.00–1.02)		(1.01–1.03)		(1.01–1.02)		(1.01–1.02)	

CI, confidence interval; BMI, body mass index; FBS, fasting blood sugar level.

*Adjusted for skipping breakfast, BMI (kg/m^2^), FBS (mg/dl), and age (years).

**Adjusted for model 1 + smoking status (none, past, vs. current), drinking ethanol amount (0–20, 20–40, 40–60, vs. over 60 g), and current treatment for hypertension, dyslipidemia, hyperuricemia, stroke, or coronary disease at their first visit during the study period.

***Adjusted for model 2+ sleep duration (<6, 6–8, vs. >8 h), exercise habit weekly (over 3 days/weeks, 1–2 days/weeks, vs. none), snacking, and late-night dinner at their first visit during the study period.

All subjects were categorized into three subgroups stratified by BMI, as follows: males, <22.2, 22.2–24.5, and ≥24.5; and females, <19.3, 19.3–21.6, and ≥21.6 kg/m^2^. The association between skipping breakfast and the prevalence of proteinuria above 1+ was evident in lower BMI subjects (BMI of males, <22.2 kg/m^2^); BMI of females, <19.3 and 19.3–21.6 kg/m^2^), even after adjusting for clinically relevant factors (model 1) and additional unhealthy behavior variables (model 2) (adjusted odds ratios of males and females: model 1, 1.67 [1.17–2.38], 1.92 [1.31–2.82], and 1.75 [1.07–2.86]; model 2, 1.66 [1.16–2.37], 1.94 [1.31–2.87], and 1.79 [1.08–2.96], respectively, whereas this association was not evident in the BMI = 22.0–24.9 and ≥25.0 kg/m^2^ subjects (**Tables 3A**, **B**).

**Table 3A:** Logistic regression analysis for the skipping breakfast and the prevalence of proteinuria above1+ in 15,875 males stratified by body mass index (BMI) levels.

Males	BMI <22.2n = 5,201 (32.8%)				22.2 ≤BMI <24.5n = 5,159 (32.5%)				24.5 ≤ BMIn = 5,515 (34.7%)			
	Univariable		Multivariable		Univariable		Multivariable		Univariable		Multivariable	
	Odds ratio (95% CI)	*p*-value	Odds ratio(95% CI)	*p*-value	Odds ratio(95% CI)	*p*-value	Odds ratio(95% CI)	*p*-value	Odds ratio(95% CI)	*p*-value	Odds ratio(95% CI)	*p*-value
**Proteinuria**												
**above 1+**			*Model 1				*Model 1				*Model 1	
**Skipping breakfast**	2.24(1.62-3.10)	<0.001	1.67(1.17-2.38)	0.004	1.65(1.13-2.39)	0.009	1.19(0.79-1.79)	0.398	1.44 (1.13-1.84)	0.004	1.29(0.99-1.68)	0.056
			**Model 21.66(1.16-2.37)	0.005			**Model 21.15(0.76-1.73)	0.505			**Model 21.29(0.98-1.68)	0.065

CI, confidence interval.

*Adjusted for age (y), BMI (kg/m^2^), FBS (mg/dL), skipping breakfast, smoking status (none, past, vs. current), drinking ethanol amount (0-20 g, 20-40 g, 40-60 g, vs. over 60 g),and current treatment for hypertension, dyslipidemia, hyperuricemia, stroke, or coronary disease at their first visit during the study period.

** Adjusted for model 1 + sleep duration (<6 hours, 6-8 hours, vs. >8 hours), exercise habit weekly (over 3 days/weeks, 1-2 days/weeks, vs. none), snacking and late night dinner at their first visit during the study period.

**Table 3B:** Logistic regression analysis for the skipping breakfast and the prevalence of proteinuria above 1+ in 11,013 females stratified by body mass index (BMI) levels.

Females		BMI <19.3n = 3,685 (33.5%)				19.3 ≤BMI <21.6n = 3,465 (31.5%)				21.6 ≤BMI n = 3,863 (35.0%)			
		Univariable		Multivariable		Univariable		Multivariable		Univariable		Multivariable	
		Odds ratio (95% CI)	*p*-value	Odds ratio(95% CI)	*p*-value	Odds ratio(95% CI)	*p*-value	Odds ratio(95% CI)	*p*-value	Odds ratio(95% CI)	*p*-value	Odds ratio(95% CI)	*p*-value
**Proteinuria**												
**above 1+**				*Model 1				*Model 1				*Model 1
**Skipping breakfast**		2.43	<0.001	1.92	0.001	2.33	<0.001	1.75	0.026	1.66	0.028	1.43	0.147
		(1.70–3.45)		(1.31–2.82)		(1.47–3.70)		(1.07–2.86)		(1.06–2.60)		(0.88–2.31)	
				**Model 2				**Model 2				**Model 2	
				1.94	0.001			1.79	0.023			1.45	0.137
				(1.31–2.87)				(1.08–2.96)				(0.89–2.34)	

CI, confidence interval.

*Adjusted for age (years), BMI (kg/m^2^), FBS (mg/dl), smoking status (none, past, vs. current), drinking ethanol amount (0–20, 20–40, 40–60, vs. over 60 g), and current treatment for hypertension, dyslipidemia, hyperuricemia, stroke, or coronary disease at their first visit during the study period.

**Adjusted for model 1 + sleep duration (<6, 6–8, vs. >8 h), exercise habit weekly (over 3 days/weeks, 1–2 days/weeks, vs. none), snacking, and late-night dinner at their first visit during the study period.

BMI is for primary analysis, and waist circumference is for further mechanistic analysis. Only 1.4% (397/26,888) of all subjects were older than 75 years. However, BMI could reflect the amount of muscle mass (22). Therefore, we performed an additional analysis on waist circumference. As shown in **Tables 4A**, **B**, males and females were divided into three groups by each level of waist circumference. Males were divided into waist circumference <81.0 (n = 4,854, 30.6%), 81.0 ≤ waist circumference <8 8.0 (n = 5,498, 34.6%), and waist circumference ≥ 88.0 cm (n = 5,523, 34.8%). In addition, females were divided into waist circumference < 73.0 (n = 3,661, 33.2%), 73.0 ≤ waist circumference < 80.0 (n = 3,532, 32.1%), and waist circumference ≥ 80.0 cm (n = 3,820, 34.7%). The association between skipping breakfast and the prevalence of proteinuria above 1+ was evident in lower waist circumference subjects (males, waist circumference < 88.0; females, waist circumference < 80.0 cm), even after adjusting for clinically relevant factors.

**Table 4A:** Logistic regression analysis for analysis for the skipping breakfast and the prevalence of proteinuria above 1+ in 15,875 males stratified by waist circumference.

Males	Waist circumference <81.0 cm4,854 (30.6%) males				81.0 ≤ Waist circumference <88.0 cm5,498 (34.6%) males				88.0 cm ≤ Waist circumference5,523 (34.8%) males			
	Univariable		Multivariable		Univariable		*Multivariable		Univariable		*Multivariable	
	Odds ratio(95% CI)	*p*-value	Odds ratio(95% CI)	*p*-value	Odds ratio(95% CI)	*p*-value	Odds ratio(95% CI)	*p*-value	Odds ratio(95% CI)	*p*-value	Odds ratio(95% CI)	*p*-value
**Proteinuria**												
**above 1+**			*Model 1				*Model 1				*Model 1	
**Skipping breakfast**	1.97	<0.001	1.49	0.029	1.96	<0.001	1.52	0.025	1.46	0.003	1.22	0.158
	(1.42-2.73)		(1.04-2.13)		(1.40-2.75)		(1.05-2.20)		(1.13-1.88)		(0.93-1.60)	
			**Model 2				**Model 2				**Model 2	
			1.46	0.039			1.51	0.030			1.19	0.218
			(1.02-2.10)				(1.04-2.19)				(0.90-1.57)	
												

CI, confidence interval.

*Adjusted for age (years), BMI (kg/m2), FBS (mg/dl), smoking status (none, past, vs. current), drinking ethanol amount (0–20, 20–40, 40–60, vs. over 60 g), and current treatment for hypertension, dyslipidemia, hyperuricemia, stroke, or coronary disease at their first visit during the study period.

**Adjusted for model 1 + sleep duration (<6 hours, 6-8 hours, vs. >8 hours), exercise habit weekly (over 3 days/weeks, 1-2 days/weeks, vs. none), snacking and late night dinner at their first visit during the study period.

**Table 4B:** Logistic regression analysis for the skipping breakfast and the prevalence of proteinuria above 1+ in 11,013 females stratified by waist circumference.

Females	Waist circumference < 73.0 cm3,661 (33.2%) females				73.0 ≤ waist circumference < 80.0 cm 3,532 (32.1%) females				80.0 cm ≤ waist circumference3,820 (34.7%) females			
	Univariable		Multivariable		Univariable		Multivariable		Univariable		Multivariable	
	Odds ratio(95% CI)	*p*-value	Odds ratio(95% CI)	*p*-value	Odds ratio(95% CI)	*p*-value	Odds ratio(95% CI)	*p*-value	Odds ratio(95% CI)	*p*-value	Odds ratio(95% CI)	*p*-value
**Proteinuria**												
**above 1+**			*Model 1				*Model 1				*Model 1	
**Skipping breakfast**	2.43	<0.001	2.08	<0.001	2.13	0.001	1.60	0.056	1.74	0.020	1.50	0.117
	(1.72–3.45)		(1.43–3.03)		(1.36–3.34)		(0.99–2.58)		(1.09–2.78)		(0.90–2.49)	
			**Model 2				**Model 2				**Model 2	
			2.08	<0.001			1.81	0.018			1.45	0.153
			(1.42–3.06)				(1.11–2.95)				(0.87–2.42)	

CI, confidence interval.

*Adjusted for age (y), waist circumference (cm), FBS (mg/dL), skipping breakfast, smoking status (none, past, vs. past, vs. current), drinking ethanol amount (0-20 g, 20-40 g, 40-60 g, vs. over 60 g), and current treatment for hypertension, hypertension, dyslipidemia, hyperuricemia, stroke, or coronary disease at their first visit during the study period.

**Adjusted for model 1 + sleep duration (<6, 6–8, vs. >8 h), exercise habit weekly (over 3 days/weeks, 1–2 days/weeks, vs. none), snacking, and late-night dinner at their first visit during the study period.

To more closely investigate the relationship between the prevalence of proteinuria and skipping breakfast, we evaluated insulin resistance (HOMA-IR) and secretory capacity (HOMA-beta). The higher the BMI or waist circumference, the higher the insulin secretion (HOMA-beta). On the other hand, the lower the BMI or waist circumference, the lower the insulin secretion (HOMA-beta) (**Supplementary Table C**).

Although we performed the same investigation and excluded the cases under treatment for diabetes, the same tendency is shown in **Supplementary Tables D–F**.

## Discussion

Previously, the prevalence of proteinuria showed a J-shaped relationship with BMI in a cross-sectional study of 62,582 asymptomatic individuals aged 20–70 years without known kidney diseases, based on the results of medical checkups (16). However, a systematic review of six studies, based on 96,175 participants and 4,935 cases, indicated that skipping breakfast was associated with an increased risk of type 2 diabetes, and the association was mediated by BMI (15). The clinical impact of BMI on the association between skipping breakfast and the prevalence of proteinuria is unclear. The present cross-sectional study identified skipping breakfast as a clinically useful indicator of proteinuria, especially in lower BMI and lower waist circumference subjects. One of the advantages of the present study was the large sample size (n = 26,888: 15,875 males and 11,013 females) and wide range of ages from 16 to 90 years (the median age of the males was 49 years, and the median age of the females was 48 years). The results of the present study may provide clinically useful evidence to establish an effective strategy to prevent the prevalence of proteinuria, which is one of the major risk factors for CKD and CVD.

Previous retrospective cohort studies reported an association between skipping breakfast and proteinuria. A retrospective cohort study including 26,764 Japanese from the general population aged ≥40 years (mean age was 68 years) showed that skipping breakfast was associated with higher risks for proteinuria onset (defined as dipstick urinary protein ≥1+) (12). Another retrospective cohort study including 5,439 females and 4,674 males, mainly young workers of one national university in Japan (median age of 31 years and 34 years, respectively), identified skipping breakfast as a significant predictor of the incidence of proteinuria (defined as dipstick urinary protein ≥1+) in females, but not in males (13).

The present study showed an additive interaction between skipping breakfast and BMI or waist circumference on the prevalence of proteinuria. The present study found that skipping breakfast was a key indicator of proteinuria, especially in lower BMI and lower waist circumference subjects.

The precise mechanism for the association between skipping breakfast and the prevalence of proteinuria is unclear; however, one of the potential explanations was the second-meal phenomenon. The second-meal phenomenon can influence the postprandial hyperglycemic response and impair insulin response at the next meal (23). The longer the fasting time, the lower the insulin concentrations, and this postprandial hyperglycemia induces pro-inflammatory cytokines (24), oxidative stress, vascular endothelial dysfunction, and proteinuria (25). In previous reports, acute glycemic changes in isolated postprandial hyperglycemia form the sharp glycemic spikes leading to endothelial dysfunction and urine albumin excretion (26). The extent of the postprandial rise in plasma glucose depends on the quantity and nature of food ingested, and the metabolic state such as loss of early insulin secretion (27) and slowed gastric emptying (28, 29). Some studies reported that lower BMI was associated with increased glycemic variability, characterized by elevated postprandial glucose excursions. This indicates that underweight or normal-weight patients have poorer beta-cell function as compared with overweight or obese patients, which may cause higher levels of postprandial blood glucose and postprandial glucose excursions in lower BMI patients (30). In our study, low insulin secretion (HOMA-beta) was shown in lower BMI and lower waist circumference subjects (**Tables 1A**, **B** and **Supplementary Table C**). This association was also shown in our investigation without the cases under treatment for diabetes (**Supplementary Tables D**, **E**). By skipping breakfast, longer fasting increases lipolysis (31). Insulin powerfully inhibits lipolysis through phosphorylation (via a PKB/Akt-dependent action) and activation of phosphodiesterase-3B (PDE-3B). This leads to the deactivation of hormone-sensitive lipase (HSL), thus decreasing the lipolytic rate (32). Therefore, lower insulin secretion leads to the deactivation of HSL and elevated free fatty acid levels. The elevated free fatty acid level involves the onset of endothelial dysfunction due to oxidative stress (33–36). However, higher BMI was associated with delayed gastric emptying (37, 38), suggesting that attenuation of postprandial hyperglycemic response at the next meal was associated with lower BMI.

In the present study, the association between proteinuria and skipping breakfast was not significant in males and females with higher BMI. Higher BMI by itself promotes the risk of proteinuria and might be less affected by skipping breakfast than lower BMI. The pathophysiology underlying the association between lower BMI and proteinuria is yet unknown. Proteinuria in lower BMI subjects might reflect the damage of the glomerulus with completely different pathophysiology as compared to vascular risk burden in obese subjects. Even if the mechanisms between lower BMI and proteinuria are not fully explained, it is widely known that the association between BMI and all-cause mortality was U-shaped (39). Furthermore, it is well known that proteinuria is a valid biomarker of increased mortality risk (40).

The present study has several limitations. First, the more detailed frequency of skipping breakfast was not identified in the present study. More information is needed to assess the optimal breakfast frequency. Second, although standardized health examinations were conducted, the present study included subjects who underwent health checkups at a single center in Japan; thus, the generalizability of the results needs to be verified in a multicenter study. Third, data on some important confounding factors were not available, such as the presence or absence of renin–angiotensin system inhibitor, shift work, and working time, which may be associated with the prevalence of proteinuria.

In conclusion, the present cross-sectional study identified an association between skipping breakfast and the prevalence of proteinuria in low BMI and low waist circumference subjects. These results suggest that lower BMI subjects with proteinuria might need to be careful about skipping breakfast. However, the direction of causality between skipping breakfast and the prevalence of proteinuria modified by BMI was unknown due to the cross-sectional study design. After this cross-sectional study, we hope a cohort study could be performed, and its efficacy should be evaluated in a randomized controlled study.

## Data availability statement

The raw data supporting the conclusions of this article will be made available by the authors, without undue reservation.

## Ethics statement

The studies involving human participants were reviewed and approved by human ethics committees of Sumitomo Hospital. Written informed consent to participate in this study was provided by the participants’ legal guardian/next of kin.

## Author contributions

JM and KK planned this study and interpreted the data. JM, TF, AM, and KaS recruited and collected clinical cases. JM conducted the statistical analysis of relevant data. JM and KK participated in the writing and modification of the article. YaT, KeS, YoT, and HR critically revised the manuscript. All authors have read and approved the manuscript. All authors contributed to the article and approved the submitted version.

## Conflict of interest

The authors declare that the research was conducted in the absence of any commercial or financial relationships that could be construed as a potential conflict of interest.

## Publisher’s note

All claims expressed in this article are solely those of the authors and do not necessarily represent those of their affiliated organizations, or those of the publisher, the editors and the reviewers. Any product that may be evaluated in this article, or claim that may be made by its manufacturer, is not guaranteed or endorsed by the publisher.
