# Supplementary material for: Lower body mass index potentiates the association between skipping breakfast and prevalence of proteinuria
Source: Front Endocrinol (Lausanne). 2022 Aug 19;13:916374. doi: 10.3389/fendo.2022.916374 (PMC9437953; doi:10.3389/fendo.2022.916374)
Supplement: Supplementary file 1 [file Table_1.pdf]

Supplement TABLE A. Clinical characteristics of 15,875 males stratified on the presence of breakfast.

Parameters	**Taking breakfast n= 12,569 (79.2%)**	**Skipping breakfast n= 3,306 (20.8%)**	***P* value**
Age (years)	51±11	44±9	<0.001
Height (cm)	170.9±6.0	171.8±6.0	<0.001
Weight (kg)	69.1±10.4	70.2±10.9	<0.001
BMI (kg/m²)	23.6±3.1	23.8±3.3	0.018
Waist circumference (cm)	85.1±8.4	85.2±9.0	0.845
**Medical History, n (%)**			
Hypertension	2,548 (20.3)	435 (13.2)	<0.001
Diabetes mellitus	1,061 (8.4)	150 (4.5)	<0.001
Dyslipidemia	2,433 (19.4)	476 (14.4)	<0.001
Stroke	189 (1.5)	22 (0.7)	<0.001
Hyperuricemia	1,225 (9.8)	244 (7.4)	<0.001
Coronary artery disease	242 (1.9)	31 (0.9)	<0.001
**Life-behavior, n (%)**			
Smoking habits			
Current smoking	3,240 (25.8)	1,537 (46.5)	<0.001
Past smoking	5,259 (41.8)	977 (29.6)	
Never	4,070 (32.4)	792 (24.0)	
Alcohol amount per day			
Over 60 g	982 (7.8)	477 (14.4)	<0.001
40-60g	2,612 (20.8)	828 (25.1)	
20-40g	3,616 (28.8)	867 (26.2)	
0-20g	5359 (42.6)	1,134 (34.3)	
Exercise habits			
Over 3 days/weeks	2,527 (20.1)	322 (9.7)	<0.001
1-2 days/weeks	4,651 (37.0)	961 (29.1)	
None	5,391 (42.9)	2,023 (61.2)	
Snacking	786 (6.3)	336 (10.2)	<0.001
Late-night dinners	5,648 (44.9)	2,101 (63.6)	<0.001
Sleeping duration (hour)			

<6 hours	4,941 (39.3)	1,520 (46.0)	<0.001
6-8 hours	7,416 (59.0)	1,743 (52.7)	
>8 hours	212 (1.7)	43 (1.3)	
**Physical findings on admission**			
Systolic blood pressure, mmHg	123±15	122±15	<0.001
Diastolic blood pressure, mmHg	78±10	76±11	<0.001
**Laboratory Data on admission**			
Hemoglobin, mg/dL	15.0±1.0	15.2±1.0	<0.001
AST, unit/L	22 (18, 26)	22 (18, 27)	0.905
ALT, unit/L	22 (16, 31)	23 (16, 34)	<0.001
Albumin, mg/dL	4.5±0.3	4.6±0.3	<0.001
Total cholesterol, mg/dL	209±33	211±36	0.029
Triglyceride, mg/dL	107 (76, 156)	116 (82, 176)	<0.001
HDL-C, mg/dL	58 (50, 69)	56 (48, 66)	<0.001
LDL-C, mg/dL	125 (106, 145)	128 (107, 150)	<0.001
FBS, mg/dL	100±20	99±19	0.050
Creatinine, mg/dL	0.84±0.10	0.83±0.11	0.040
Uric acid, mg/dL	6.2±1.2	6.4±1.3	<0.001
eGFR, mL/min/1.73m^2	76.7 (69.6, 85.2)	80.4 (73.0, 89.6)	<0.001
Hemoglobin A1c (NGSP), %	5.6 (5.4, 5.8)	5.5 (5.2, 5.7)	<0.001
HOMA-beta	58.1 (40.5, 82.1)	62.7 (44.4, 90.0)	<0.001
HOMA-IR	1.30 (0.88, 1.93)	1.38 (0.93, 2.08)	<0.001
Proteinuria above (1+)	446 (3.6)	197 (6.0)	<0.001

Note: Categorical variables are expressed as numbers (percentages) and continuous variables are shown as mean ± standard deviation or median (interquartile range), as appropriate.

Abbreviations: BMI, body mass index; ALT, alanine aminotransferase; AST, aspartate transaminase; HDL, high-density lipoprotein; LDL, low-density lipoprotein; FBS, fasting blood sugar level; eGFR, estimated glomerular filtration rate.
