# Supplementary material for: Lower body mass index potentiates the association between skipping breakfast and prevalence of proteinuria
Source: Front Endocrinol (Lausanne). 2022 Aug 19;13:916374. doi: 10.3389/fendo.2022.916374 (PMC9437953; doi:10.3389/fendo.2022.916374)
Supplement: Supplementary file 2 [file Table_2.pdf]

Supplement TABLE B. Clinical characteristics of 11,013 females stratified on the presence of breakfast.

Parameters	**Taking breakfast n= 9,499 (86.3%)**	**Skipping breakfast n= 1,514 (13.8%)**	***P* value**
Age (years)	49±11	43±11	<0.001
Height (cm)	158.2±5.6	159.0±5.6	<0.001
Weight (kg)	52.6±8.3	53.0±9.1	0.640
BMI (kg/m²)	21.0±3.2	21.0±3.4	0.066
Waist circumference (cm)	77.3±8.9	76.9±9.4	0.006
**Medical History, n (%)**			
Hypertension	889 (9.4)	79 (5.2)	<0.001
Diabetes mellitus	228 (2.4)	20 (1.3)	0.009
Dyslipidemia	1,215 (12.8)	116 (7.7)	<0.001
Stroke	70 (0.7)	8 (0.5)	0.369
Hyperuricemia	16 (0.2)	2 (0.1)	0.745
Coronary artery disease	242 (1.9)	31 (0.9)	0.024
**Life-behavior, n (%)**			
Smoking habits			
Current smoking	547 (5.8)	333 (22.0)	<0.001
Past smoking	1,000 (10.5)	268 (17.7)	
Never	7,952 (83.7)	913 (60.3)	
Alcohol amount per day			
Over 60 g	111 (1.2)	89 (5.9)	<0.001
40-60g	427 (4.5)	169 (11.2)	
20-40g	1,213 (12.8)	303 (20.2)	
0-20g	7,748 (81.6)	953 (63.0)	
Exercise habits			
Over 3 days/weeks	1,678 (17.7)	1,052 (69.5)	<0.001
1-2 days/weeks	2,559 (26.9)	328 (21.7)	
None	5,262 (22.8)	353 (23.3)	
Snacking	2,164 (22.8)	353 (23.3)	0.646
Late-night dinners	1,728 (18.2)	630 (41.6)	<0.001
Sleeping duration (hour)			

<6 hours	3,763 (39.6)	604 (39.9)	0.007
6-8 hours	5,557 (58.5)	863 (57.0)	
>8 hours	179 (1.9)	47 (3.1)	
**Physical findings on admission**			
Systolic blood pressure, mmHg	116±17	113±15	<0.001
Diastolic blood pressure, mmHg	72±11	70±10	<0.001
**Laboratory Data on admission**			
Hemoglobin, mg/dL	13.0±1.2	13.0±1.2	0.933
AST, unit/L	19 (16, 22)	17 (15, 21)	<0.001
ALT, unit/L	14 (11, 19)	13 (10, 17)	<0.001
Albumin, mg/dL	4.4±0.3	4.4±0.3	0.697
Total cholesterol, mg/dL	213±37	206±39	<0.001
Triglyceride, mg/dL	71 (53, 99)	71 (52, 101)	0.803
HDL-C, mg/dL	73 (63, 84)	72 (62, 83)	0.097
LDL-C, mg/dL	117 (97, 140)	111 (91, 134)	<0.001
FBS, mg/dL	92±13	91±11	0.012
Creatinine, mg/dL	0.62±0.08	0.62±0.09	0.098
Uric acid, mg/dL	4.4±0.9	4.5±1.1	0.030
e-GFR, mL/min/1.73m^2	80.0 (71.8, 89.7)	83.5 (74.6, 94.5)	<0.001
Hemoglobin A1c (NGSP), %	5.5 (5.2, 5.7)	5.4 (5.1, 5.6)	<0.001
HOMA-beta	63.3 (46.7, 86.1)	65.1 (47.1, 90.0)	0.028
HOMA-IR	1.03 (0.73, 1.49)	1.03 (0.72, 1.50)	0.716
Proteinuria above (1+)	290 (3.1)	97 (6.4)	<0.001

Note: Categorical variables are expressed as numbers (percentages) and continuous variables are shown as mean ± standard deviation or median (interquartile range), as appropriate.

Abbreviations: BMI, body mass index; ALT, alanine aminotransferase; AST, aspartate transaminase; HDL, high-density lipoprotein; LDL, low-density lipoprotein; FBS, fasting blood sugar level; eGFR, estimated glomerular filtration rate.
