# Supplementary material for: Lower body mass index potentiates the association between skipping breakfast and prevalence of proteinuria
Source: Front Endocrinol (Lausanne). 2022 Aug 19;13:916374. doi: 10.3389/fendo.2022.916374 (PMC9437953; doi:10.3389/fendo.2022.916374)
Supplement: Supplementary file 3 [file Table_3.pdf]

Supplement TABLE C. The clinical characteristics of 15,875 males and 11,013 females stratified by the presence of breakfast, body mass index (BMI) or waist circumference.

**Male**	**BMI <22.2**		**22.2 ≤BMI <24.5**		**24.5 ≤ BMI**	
	Skipping breakfast (n=1,089)	Taking breakfast (n=4,112)	Skipping breakfast (n=970)	Taking breakfast (n=4,189)	Skipping breakfast (n=1,247)	Taking breakfast (n=4,268)
**Proteinuria above 1+**	61 (9.5)	106 (16.5)	39 (6.1)	104 (16.2)	97 (15.1)	236 (36.7)
**HOMA-beta**	50.4 (37.0, 68.4)	47.0 (34.0, 64.3)	60.6 (45.0, 84.0)	57.7 (41.0, 78.4)	79.2 (56.3, 113.1)	73.2 (51.8, 104.0)
**HOMA-IR**	0.98 (0.68, 1.30)	0.92 (0.66, 1.27)	1.34 (0.97, 1.85)	1.28 (0.92, 1.75)	2.01 (1.42, 3.00)	1.89 (1.32, 2.74)
	**Waist circumference (cm) <81.0**		**81.0 ≤ Waist circumference (cm) <88.0**		**88.0 ≤ Waist circumference (cm)**	
	Skipping breakfast (n=1,035)	Taking breakfast (n=3,819)	Skipping breakfast (n=1,080)	Taking breakfast (n=4,418)	Skipping breakfast (n=1,191)	Taking breakfast (n=4,332)
**Proteinuria above 1+**	57 (5.5)	110 (2.9)	51 (4.7)	109 (2.5)	89 (7.5)	227 (5.2)
**HOMA-beta**	49.5 (37.1, 67.6)	47.2 (34.0, 65.6)	62.1 (45.9, 85.7)	56.9 (40.8, 78.5)	79.2 (55.8, 113.5)	72.0 (51.2, 101.8)
**HOMA-IR**	0.93 (0.67, 1.26)	0.90 (0.64, 1.23)	1.37 (1.00, 1.89)	1.27 (0.91, 1.74)	2.05 (1.45, 3.06)	1.90 (1.34, 2.74)

**Female**	**BMI <19.3**		**19.3 ≤BMI <21.6**		**21.6 ≤ BMI**	
	Skipping breakfast (n=559)	Taking breakfast (n=3,126)	Skipping breakfast (n=431)	Taking breakfast (n=3,034)	Skipping breakfast (n=524)	Taking breakfast (n=3,339)
**Proteinuria above 1+**	47 (12.1)	114 (29.5)	25 (6.5)	78 (20.2)	25 (6.5)	98 (25.3)
**HOMA-beta**	60.0 (42.0, 80.1)	56.0 (41.7, 74.7)	63.0 (47.5, 86.4)	62.6 (46.3, 83.6)	76.6 (55.9, 104.0)	72.0 (54.0, 99.5)
**HOMA-IR**	0.81 (0.60, 1.09)	0.81 (0.60, 1.12)	0.99 (0.71, 1.30)	0.98 (0.72, 1.33)	1.46 (1.00, 2.16)	1.41 (0.99, 2.03)
	**Waist circumference (cm) <73.0**		**73.0 ≤ Waist circumference (cm) <80.0**		**80.0 ≤ Waist circumference (cm)**	
	Skipping breakfast (n=556)	Taking breakfast (n=3,105)	Skipping breakfast (n=456)	Taking breakfast (n=3,076)	Skipping breakfast (n=502)	Taking breakfast (n=3,318)
**Proteinuria above 1+**	48 (8.6)	116 (3.7)	26 (5.7)	85 (2.8)	23 (4.6)	89 (2.7)
**HOMA-beta**	60.5 (42.7, 81.4)	57.3 (42.4, 76.5)	64.1 (47.2, 86.9)	61.3 (46.0, 82.6)	74.7 (53.5, 101.6)	72.0 (53.2, 99.0)
**HOMA-IR**	0.80 (0.60, 1.10)	0.81 (0.61, 1.13)	1.02 (0.71, 1.44)	0.98 (0.71, 1.33)	1.37 (0.97, 2.13)	1.41 (0.99, 2.03)

Abbreviations: BMI, body mass index
