# Supplementary material for: Lower body mass index potentiates the association between skipping breakfast and prevalence of proteinuria
Source: Front Endocrinol (Lausanne). 2022 Aug 19;13:916374. doi: 10.3389/fendo.2022.916374 (PMC9437953; doi:10.3389/fendo.2022.916374)
Supplement: Supplementary file 4 [file Table_4.pdf]

Supplement TABLE D. Logistic regression analysis for the skipping breakfast and the prevalence of proteinuria above1+ in 15,249 males without the cases under treatment for diabetes stratified by body mass index (BMI) levels.

	**BMI <22.2<br>n = 5,065 (33.2%)**				**22.2 ≤BMI <24.5<br>n = 4,974 (32.6%)**				**24.5 ≤ BMI<br>n = 5,210 (34.2%)**			
	Univariable		*Multivariable		Univariable		*Multivariable		Univariable		*Multivariable	
	Odds ratio (95% CI)	*P* value	Odds ratio (95% CI)	*P* value	Odds ratio (95% CI)	*P* value	Odds ratio (95% CI)	*P* value	Odds ratio (95% CI)	*P* value	Odds ratio (95% CI)	*P* value
**Proteinuria above 1+**												
**Skipping breakfast**	2.32 (1.67-3.22)	<0.001	***model 1** 1.65 (1.15-2.36)	0.006	1.69 (1.16-2.47)	0.006	***model 1** 1.17 (0.78-1.76)	0.447	1.51 (1.16-1.96)	0.002	***model 1** 1.28 (0.97-1.69)	0.081
			****model 2** 1.62 (1.13-2.33)	0.009			****model 2** 1.13 (0.75-1.71)	0.558			****model 2** 1.28 (0.97-1.69)	0.083

Abbreviations: CI, confidence interval.

* Adjusted for age (y), BMI (kg/m^2), FBS (mg/dL), smoking status (none, past, vs. current), drinking ethanol amount (0-20 g, 20-40 g, 40-60 g, vs. over 60 g), sleep duration (< 6 hours, 6-8 hours, vs. >8 hours) and current treatment for hypertension, dyslipidemia, hyperuricemia, stroke, or coronary disease at their first visit during the study period. ** Adjusted for model 1 + sleep duration (<6 hours, 6-8 hours, vs. >8 hours), exercise habit weekly (over 3 days/weeks, 1-2 days/weeks, vs. none), snacking and late night dinner at their first visit during the study period.
