# Supplementary material for: Lower body mass index potentiates the association between skipping breakfast and prevalence of proteinuria
Source: Front Endocrinol (Lausanne). 2022 Aug 19;13:916374. doi: 10.3389/fendo.2022.916374 (PMC9437953; doi:10.3389/fendo.2022.916374)
Supplement: Supplementary file 5 [file Table_5.pdf]

Supplement TABLE E. Logistic regression analysis for the skipping breakfast and the prevalence of proteinuria above 1+ in 10,894 females without the cases under treatment for diabetes stratified by body mass index (BMI) levels.

	**BMI <19.3 n = 3,670 (33.7%)**				**19.3 ≤BMI <21.6 n = 3,442 (31.6%)**				**21.6 ≤ BMI n = 3,782 (34.7%)**			
**Skipping breakfast**	Univariable		*Multivariable		Univariable		*Multivariable		Univariable		*Multivariable	
	Odds ratio (95% CI)	*P* value	Odds ratio (95% CI)	*P* value	Odds ratio (95% CI)	*P* value	Odds ratio (95% CI)	*P* value	Odds ratio (95% CI)	*P* value	Odds ratio (95% CI)	*P* value
**Proteinuria above 1+**												
**Skipping breakfast**	2.44 (1.71-3.47)	<0.001	***model 1** 1.92 (1.30-2.82)	0.001	2.36 (1.49-3.75)	<0.001	*** model 1** 1.75 (1.07-2.86)	0.025	1.59 (1.00-2.54)	0.051	*** model 1** 1.37 (0.83-2.26)	0.215
			** *model 2** 1.93 (1.31-2.85)	0.001			** *model 2** 1.81 (1.10-3.00)	0.020			** *model 2** 1.39 (0.84-2.30)	0.203

Abbreviations: CI, confidence interval.

* Adjusted for age (y), BMI (kg/m^2), FBS (mg/dL), smoking status (none, past, vs. current), drinking ethanol amount (0-20 g, 20-40 g, 40-60 g, vs. over 60 g), sleep duration (< 6 hours, 6-8 hours, vs. >8 hours) and current treatment for hypertension, dyslipidemia, hyperuricemia, stroke, or coronary disease at their first visit during the study period. ** Adjusted for model 1 + sleep duration (<6 hours, 6-8 hours, vs. >8 hours), exercise habit weekly (over 3 days/weeks, 1-2 days/weeks, vs. none), snacking and late night dinner at their first visit during the study period.
