# Supplementary material for: Lower body mass index potentiates the association between skipping breakfast and prevalence of proteinuria
Source: Front Endocrinol (Lausanne). 2022 Aug 19;13:916374. doi: 10.3389/fendo.2022.916374 (PMC9437953; doi:10.3389/fendo.2022.916374)
Supplement: Supplementary file 6 [file Table_6.pdf]

Supplement TABLE F. The clinical characteristics of 15,249 males and 10,894 females without the cases under treatment for diabetes stratified by the presence of breakfast, body mass index (BMI) or waist circumference.

**Male**	**BMI <22.2**		**22.2 ≤BMI <24.5**		**24.5 ≤ BMI**	
	Skipping breakfast (n=1,084)	Taking breakfast (n=3,981)	Skipping breakfast (n=959)	Taking breakfast (n=4,015)	Skipping breakfast (n=1,202)	Taking breakfast (n=4,008)
**Proteinuria above 1+**	60 (5.5)	98 (2.5)	39 (4.1)	98 (2.4)	87 (7.2)	197 (4.9)
**HOMA-beta**	50.7 (37.1, 68.5)	47.7 (34.9, 65.3)	60.8 (45.5, 84.5)	59.0 (42.8, 79.6)	79.7 (57.5, 114.4)	75.1 (54.0, 105.7)
**HOMA-IR**	0.98 (0.68, 1.30)	0.92 (0.66, 1.26)	1.34 (0.97, 1.84)	1.27 (0.91, 1.73)	1.99 (1.41, 2.92)	1.85 (1.31, 2.67)
	**Waist circumference (cm) <81.0**		**81.0 ≤ Waist circumference (cm) <88.0**		**88.0 ≤ Waist circumference (cm)**	
	Skipping breakfast (n=1,035)	Taking breakfast (n=3,819)	Skipping breakfast (n=1,080)	Taking breakfast (n=4,418)	Skipping breakfast (n=1,191)	Taking breakfast (n=4,332)
**Proteinuria above 1+**	57 (5.5)	110 (2.9)	51 (4.7)	109 (2.5)	89 (7.5)	227 (5.2)
**HOMA-beta**	49.5 (37.1, 67.6)	47.2 (34.0, 65.6)	62.1 (45.9, 85.7)	56.9 (40.8, 78.5)	79.2 (55.8, 113.5)	72.0 (51.2, 101.8)
**HOMA-IR**	0.93 (0.67, 1.26)	0.90 (0.64, 1.23)	1.37 (1.00, 1.89)	1.27 (0.91, 1.74)	2.05 (1.45, 3.06)	1.90 (1.34, 2.74)

**Female**	**BMI <19.3**		**19.3 ≤BMI <21.6**		**21.6 ≤ BMI**	
	Skipping breakfast (n=559)	Taking breakfast (n=3,111)	Skipping breakfast (n=429)	Taking breakfast (n=3,013)	Skipping breakfast (n=516)	Taking breakfast (n=3,266)
**Proteinuria above 1+**	47 (8.4)	113 (3.6)	25 (5.8)	77 (2.6)	23 (4.5)	93 (2.9)
**HOMA-beta**	60.0 (42.0, 80.1)	56.0 (42.0, 74.8)	63.0 (47.6, 86.4)	63.0 (46.6, 84.0)	77.3 (56.0, 104.4)	73.0 (54.6, 99.7)
**HOMA-IR**	0.81 (0.60, 1.09)	0.81 (0.60, 1.12)	0.99 (0.72, 1.30)	0.98 (0.72, 1.33)	1.44 (1.00, 2.11)	1.40 (0.98, 2.00)
	**Waist circumference (cm) <73.0**		**73.0 ≤ Waist circumference (cm) <80.0**		**80.0 ≤ Waist circumference (cm)**	
	Skipping breakfast (n=556)	Taking breakfast (n=3,105)	Skipping breakfast (n=456)	Taking breakfast (n=3,076)	Skipping breakfast (n=502)	Taking breakfast (n=3,318)
**Proteinuria above 1+**	48 (8.6)	116 (3.7)	26 (5.7)	85 (2.8)	23 (4.6)	89 (2.7)
**HOMA-beta**	60.5 (42.7, 81.4)	57.3 (42.4, 76.5)	64.1 (47.2, 86.9)	61.3 (46.0, 82.6)	74.7 (53.5, 101.6)	72.0 (53.2, 99.0)
**HOMA-IR**	0.80 (0.60, 1.10)	0.81 (0.61, 1.13)	1.02 (0.71, 1.44)	0.98 (0.71, 1.33)	1.37 (0.97, 2.13)	1.41 (0.99, 2.03)
